# Harnessing the Power of PAOO and Invisalign: An Interdisciplinary Approach to Orthodontic Care

**DOI:** 10.3390/medicina59050987

**Published:** 2023-05-20

**Authors:** Kayvon Javid, Rafael Coutinho Mello-Machado, Pietro Montemezzi, Rodrigo dos Santos Pereira, Adam Lowenstein, Carlos Fernando Mourão

**Affiliations:** 1 Department of Oral Surgery, School of Dentistry, Fluminense Federal University, Niterói 24020-140, Brazil; onecure@aol.com; 2 Department of Implant Dentistry, Universidade Iguaçu, Nova Iguaçu, Rio de Janeiro 26260-045, Brazil; rafaelcoutinhodemello@yahoo.com.br; 3 Department of Dentistry, San Raffaele Hospital, 20132 Milan, Italy; 4 Department of Oral & Maxillofacial Surgery, University of Grande Rio-UNIGRANRIO, Rio de Janeiro 25071-202, Brazil; 5 Department of Periodontology, Dental Research Administration, Tufts University School of Dental Medicine, Boston, MA 02111, USA

**Keywords:** orthodontics, corticotomy, dental surgery, oral surgery

## Abstract

The present article explores the benefits of combining periodontally accelerated osteogenic orthodontics (PAOO) with Invisalign for optimal orthodontic treatment outcomes. PAOO is an interdisciplinary dental technique that minimizes complications and accelerates tooth movement while enhancing various orthodontic treatments. In conjunction with Invisalign, PAOO provides a discreet and comfortable solution for patients seeking to improve their smile. The study presents two challenging cases successfully treated using this combined approach, emphasizing the technique’s potential to reduce treatment times and improve orthodontic outcomes. The interdisciplinary approach of PAOO ensures long-term success and stability by preserving periodontal structures and addressing potential bony defects. By incorporating bone grafting materials, PAOO helps prevent common concerns in traditional orthodontic treatments, such as bony defects and gingival recession. Furthermore, the combination with Invisalign offers a more aesthetically pleasing and comfortable treatment experience, allowing patients to maintain their self-esteem and confidence throughout the treatment. Despite the potential advantages, dental professionals must manage patient expectations and address potential complications to ensure the best possible results. Overall, the integration of PAOO and Invisalign demonstrates a viable alternative for patients who do not want to proceed with orthognathic surgery, enhancing patient satisfaction and overall treatment outcomes.

## 1. Introduction

Periodontally accelerated osteogenic orthodontics (PAOO) is a dental technique that has evolved from the corticotomy-assisted orthodontic treatment (CAOT) concept [1]. PAOO combines selective decortication bone activation with orthodontic forces, revolutionizing dentistry by providing personalized treatment options that minimize complications and improve overall outcomes. The interdisciplinary approach of PAOO reduces the risk of root resorption, relapses, decay, and infection while accelerating tooth movement and enhancing various orthodontic treatments [1,2].

Considering the lack of clinical studies related to PAOO in the literature, it is important to consider well-known treatments (e.g., orthognathic surgery) before choosing this elective approach. However, the significance of PAOO lies in its ability to offer better treatment outcomes for patients. By using bone grafting materials in conjunction with corticotomy procedures, PAOO aims to prevent adverse outcomes such as bony defects, gingival recession, and potential damage to the periodontal structures [3,4,5]. In addition, PAOO is an effective technique that significantly accelerates tooth movement, making it particularly advantageous for adult patients who desire to avoid lengthy ortho-surgical procedures. This method yields optimal results by stimulating the alveolar bone remodeling process and enhancing the biological response, leading to faster tooth realignment and overall shorter treatment duration. Patients highly value this expedited approach as it minimizes the impact of prolonged treatment on their personal and professional lives [2].

PAOO’s interdisciplinary approach provides numerous patient benefits, including accelerated treatment times, improved orthodontic outcomes, and minimized complications. PAOO focuses on preserving periodontal structures and addressing potential bony defects, ensuring orthodontic treatments’ long-term success and stability [2]. PAOO can also be combined with invisible aligners, such as Invisalign, to provide a more aesthetically pleasing and comfortable experience for the patient [6,7]. Integrating advanced orthodontic technologies expedites treatment and offers a discreet solution for individuals seeking to improve their smile with minimal impact on their daily lives [7].

This study aims to provide reliable insights into the potential benefits of combining advanced orthodontic technologies to achieve optimal treatment outcomes. The authors utilized the PAOO and Invisalign technologies to offer customized treatment options that minimized complications and improved overall outcomes. The study presents two challenging cases successfully treated using the PAOO technique in conjunction with Invisalign. The findings of these reports will contribute significantly to the advancement of orthodontic treatment, offering valuable information to clinicians seeking to provide their patients with the most effective treatment options.

## 2. Case Reports

The authors of this study have followed the CARE (CAse REport) guidelines [8] to ensure a thorough and transparent reporting of their clinical and radiographic findings. By adhering to the CARE checklist and recommendations, they have collected and reported data systematically, which increases the reliability and generalizability of their findings. This approach to data collection, analysis, and reporting enhances scientific knowledge and enables future research to replicate and validate their results.

The two cases followed the same procedure, but the patients differed in age—one was 26, and the other was 34. The 26-year-old woman had an anterior open bite, while the 34-year-old woman had a Class III malocclusion (according to Angle’s Classification) and a severe crossbite (supplemental files). Both patients had previously undergone evaluations with cephalometric radiography and tomography. NemoFAB Version 2.1 (NEMOTEC software, Madrid, Spain) was used to evaluate the patients, which led to the suggestion of orthognathic surgery as the initial ortho-surgical approach. However, periodontally accelerated osteogenic orthodontics (PAOO) was also presented as an alternative treatment option. 

Before the start of the treatment process, the dental team made sure to inform both patients of the proposed treatment plans. They went into great detail in explaining all the possible risks, benefits, and outcomes associated with each procedure. This was to ensure that the patients had a clear understanding of what to expect every step of the way throughout their treatment.

**Figure 1:**
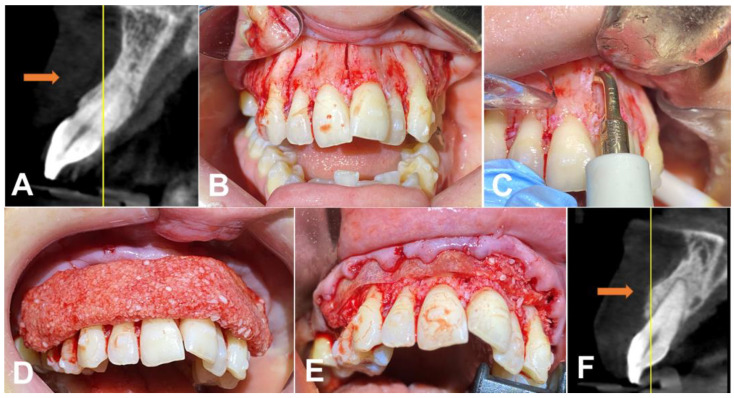
(**A**) Initial representative image of the CBCT before the bone graft, with an arrow indicating the thickness of the wall of the bone.; (**B**) cuts in the cortical bone between the teeth; (**C**) deep approach using the surgical tip in the palatal bone raphe; (**D**) bone graft (sticky bone) placed on top of the native bone after decortication and cuts; (**E**) membrane placed on top of the graft; and (**F**) representative image of the bone wall one year after treatment (arrow).

Both patients were given the opportunity to ask questions and seek clarification on any concerns they may have had. After doing so, they willingly agreed to proceed with their respective treatment plans. The dental team took all the necessary steps to obtain formal agreement from the patients, ensuring that the process was thorough and complete. This approach was taken to ensure that the patients fully understood the consequences of their decisions and were confident in their choices. The patients underwent a PAOO procedure to correct the malocclusion. Before the surgery, the patients underwent a cone beam computed tomography (CBCT) scan to assess their bone structure so a customized Invisalign treatment plan could be developed (Figure 1A). During the surgery, a piezoelectric motor (Piezosurgery^®^, Piezosurgery Inc., a Mectron Company, Columbus, OH, USA) with a straight tip (OT12) was utilized to create precise cuts in the cortical bone between the teeth. The cuts extended approximately 5 mm to the middle of the palatal bone raphe (Figure 1B,C). They were followed by bone decortication using a diamond tip (OT1). After making the cuts between the teeth, an allograft bone graft (Maxxeus^®^, Dayton, OH, USA) was placed over the area to enhance and strengthen the bone structure. In addition, an allograft membrane/barrier was placed on top of the bone graft to protect the area.

The bone graft was prepared using leucocyte–platelet-rich fibrin (L-PRF) [9,10]. In order to produce L-PRF, both red and white cap tubes were utilized to centrifuge the patient’s blood using a machine manufactured by Biohorizons^®^ (AL, USA). Taking into account the centrifuge’s balance and the types of tubes used, a single centrifugation was performed to simultaneously produce the L-PRF membrane and Liquid L-PRF, following the “One Spin—Double Process” (a method by C.F.M.) (L-PRF protocol: ~700 RCF-max (~400 RCF-clot) for 8 min) [9,10]. After producing the L-PRF, the membranes were cut into smaller pieces and mixed with the bone graft. Subsequently, the Liquid L-PRF was incorporated into the graft, and after a waiting period of 4 to 6 min, the clot was integrated into the bone substitute, creating the “Sticky Bone” [9,10]. (Figure 1D), which facilitated the handling of the graft material. Following the bone graft and membrane (Pericardium, Maxxeus^®^, OH, USA) placement (Figure 1F) on top of the pericardium membrane, the L-PRF membrane was placed, and primary closure was achieved using Polytetra Fluoro Ethylene (PTFE) 4–0 sutures (Ethicon^®^, Johnson & Johnson, New Brunswick, NJ, USA).

Both were prescribed Augmentin 625 mg (amoxicillin and clavulanate) every 12 h for seven days and Naproxen Sodium 220 mg every 12 h for five days to manage pain and prevent infection. In addition to the medications, the patients were instructed to maintain proper oral hygiene by using a Chlorhexidine 0.12% mouthwash twice daily for 14 days. After 10 days, the patients were scheduled to have their sutures removed. Suture removal is an important step in the postoperative care process, as it ensures that the healing tissues have had ample time to knit together and regain strength. In addition, removing the sutures at the appropriate time minimizes the risk of complications such as wound dehiscence or infection, and the patients can progress toward full recovery.

**Figure 2:**
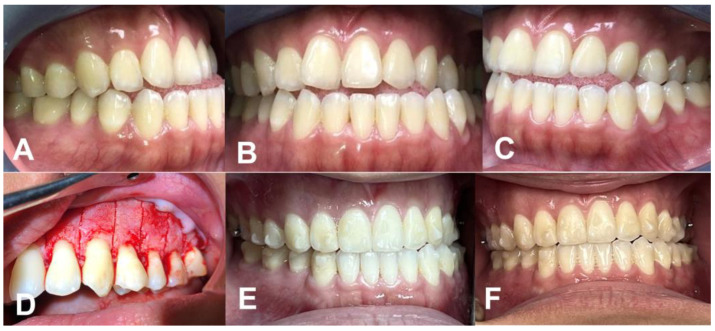
(**A**) Lateral (right side), (**B**) frontal, (**C**) left side views; (**D**) surgical procedure, periodontally accelerated osteogenic orthodontics; (**E**) lateral, and (**F**) frontal views after one-year follow-up.

**Figure 3:**
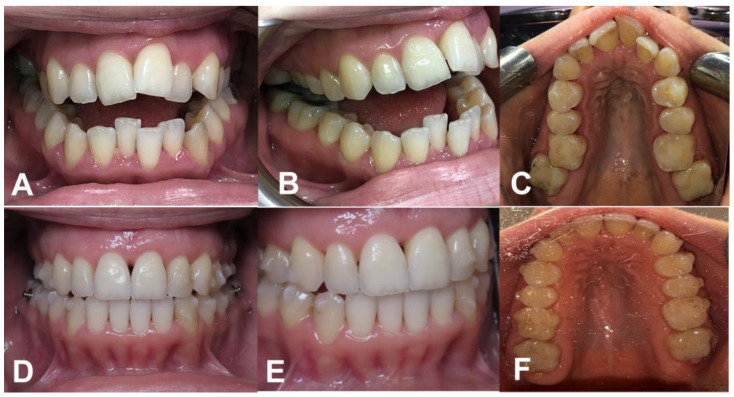
(**A**)Frontal, (**B**) lateral, and (**C**) occlusal views before the treatment. (**D**) Frontal, (**E**) lateral, and (**F**) occlusal views after one-year follow-up.

After the surgical procedure, they began using the first Invisalign (Invisalign^®^, La Puente, CA, USA) tray and changed trays weekly for a total treatment period of 12 months. This orthodontic intervention successfully corrected the present cases, showcasing the effectiveness of the PAOO procedure in combination with Invisalign therapy (Figure 2 and Figure 3).

Both patients must undergo regular check-ups to ensure the effectiveness of their treatment in the long run. Dental professionals will consistently monitor their orthodontic corrections to prevent any potential relapse and assess their stability. Furthermore, the patients have been educated on the critical importance of maintaining proper oral hygiene practices and receiving regular dental cleanings to uphold optimal oral health.

## 3. Discussion

Periodontally accelerated osteogenic orthodontics (PAOO) provides a major edge to patients seeking to fix dental irregularities without resorting to orthognathic surgery and modifying their facial appearance. Its primary advantage lies in its capacity to accelerate tooth movement, which proves particularly advantageous for adult patients worried about the duration of ortho-surgical procedures.

The interdisciplinary approach of PAOO allows for enhanced resolution of dental issues such as crowding, impacted teeth, open bites, and molar intrusion. Combining PAOO with Invisalign provides patients with a more aesthetically pleasing and comfortable treatment option. Invisalign aligners are discreet and removable, enabling patients to maintain their self-esteem and confidence throughout the treatment [7]. Additionally, using Invisalign aligners can make it easier for patients to maintain proper oral hygiene during treatment, reducing the risk of dental decay and gum disease.

A review conducted recently [11] has highlighted that PAOO is an attractive option for certain patients due to its numerous benefits. One significant advantage of this technique is that it enhances the volume of the alveolar bone and periodontium, allowing for the correction of dehiscences and fenestrations. Moreover, PAOO is known to reduce treatment time, providing 3–4-times-faster active orthodontic treatment when compared to traditional methods. This technique also offers better post-treatment stability and a lower risk of relapse, extending the scope of malocclusion treatment, and in some cases, avoiding orthognathic surgery and extractions. PAOO can also be utilized in some cases to improve a patient’s profile if needed and facilitate the rapid recovery of impacted teeth, such as canines. In both cases discussed in this report, the benefits of this technique were observed.

Additionally, bone grafting materials are used in PAOO procedures; this helps to maintain the integrity of the periodontal structures and prevent issues such as bony defects and gingival recession, which are often seen in traditional orthodontic treatments. This approach also helps to ensure the overall health of the periodontium, which is crucial for the long-term success and stability of orthodontic treatment outcomes [12].

While PAOO has its benefits, it may not be appropriate for all patients due to certain contraindications [11]. Individuals with thinner mandibular cortices may have a higher risk of complications and should avoid this treatment. Patients who have active periodontal disease or gingival recession should also steer clear of PAOO as it could worsen their existing conditions. PAOO is not recommended for palatal expansion or in the treatment of severe posterior cross-bite since it may not yield the desired results. Lastly, it is not advisable to use PAOO for bimaxillary protrusion accompanied by a gummy smile. In such cases, alternative treatment options may be more effective in achieving optimal outcomes.

It is crucial to consider any possible negative effects and complications when receiving treatment [2,3,11,12,13]. An aesthetic issue that may occur is a black space between the incisors after treatment, as previously mentioned. Despite this, the patient expressed satisfaction with the outcome, highlighting the importance of keeping patients informed and addressing their concerns throughout the treatment process. In the second scenario discussed, the authors underwent palatal expansion. However, this is not the usual method used to address such cases. The authors suggest conducting further clinical studies to provide more insight into the matter.

Dental professionals can ensure that patients make informed decisions about their care by thoroughly explaining the treatment plan, potential risks, and benefits. Furthermore, proper post-treatment follow-up and maintenance can help identify and address potential complications or concerns, ensuring patients achieve the most favorable results and long-term satisfaction with their treatment.

The presented surgical technique aims to offer an ortho-surgical alternative in treating patients with dentofacial deformities. Nevertheless, it does not eliminate the surgical and compensatory treatments documented in the literature. It is essential to consider the patients’ preferences, as both the selection and conclusion of treatment are influenced by their opinions. Consequently, it is imperative to conduct future clinical studies (e.g., evaluation of different ortho-surgical approaches) that compare the available options while considering the individual considerations of each patient.

## 4. Conclusions

Combined with Invisalign, periodontally accelerated osteogenic orthodontics (PAOO) offers a viable alternative to orthognathic surgery for patients seeking to correct dental disorders. With the potential to accelerate treatment times, preserve periodontal structures, and provide a more discreet and comfortable treatment experience, PAOO and Invisalign have the potential to enhance patient satisfaction and improve overall treatment outcomes. Recognizing these promising aspects, the authors of the present article encourage further clinical studies to substantiate and refine the use of the PAOO technique in orthodontic practice. However, it is crucial for dental professionals to manage patient expectations and address potential complications to ensure the best possible results.

## Data Availability

The data that support the findings of this study are available on request from the corresponding author.

## Supplementary Materials

The following supporting information can be downloaded at: https://www.mdpi.com/article/10.3390/medicina59050987/s1, Figure S1: Cephalometric analysis patient 1; Figure S2: Cephalometric analysis patient 2; Videos S: Invisalign^®^ System - ClinCheck Pro 6.0^®^ software (patient 1 and 2).
