# Supplementary figures and images for: Harnessing the Power of PAOO and Invisalign: An Interdisciplinary Approach to Orthodontic Care
Source: Medicina (Kaunas). 2023 May 20;59(5):987. doi: 10.3390/medicina59050987 (PMC10221098; doi:10.3390/medicina59050987)

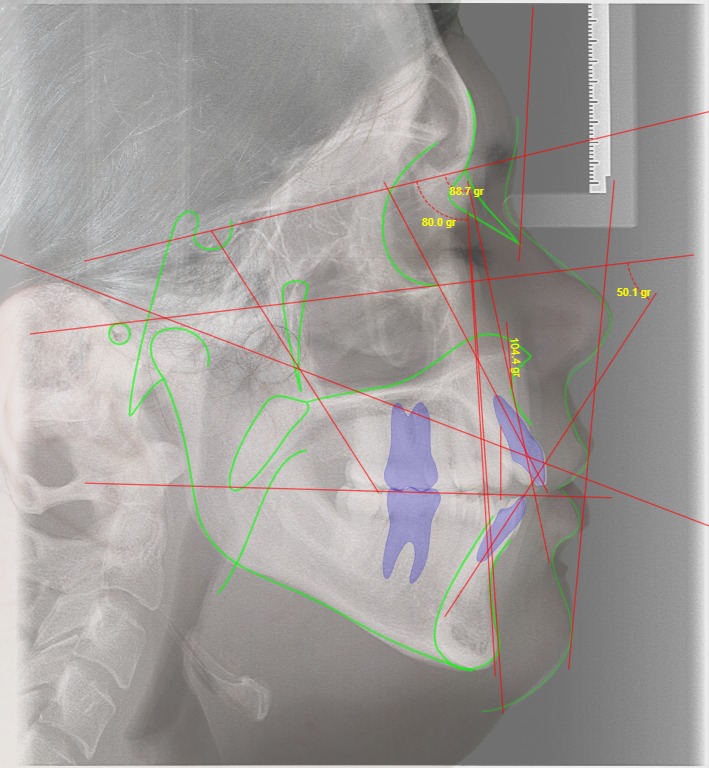

Supplement: Supplementary file 1 [file medicina-59-00987-s001.zip › Patient 1 .jpeg]

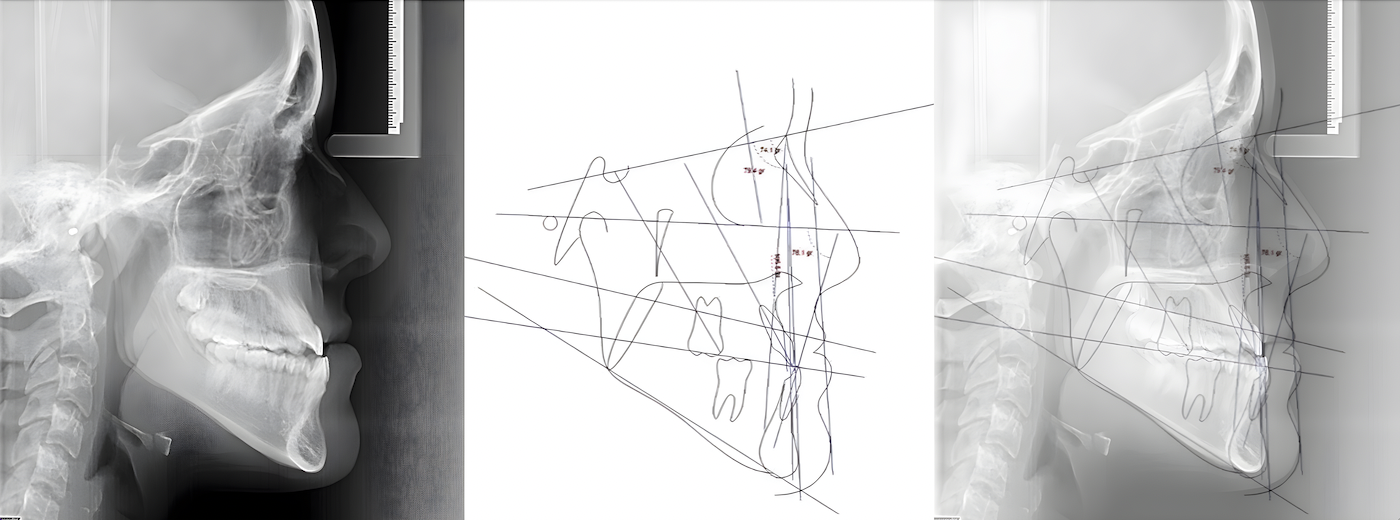

Supplement: Supplementary file 1 [file medicina-59-00987-s001.zip › Patient 2.png]
